# Supplementary material for: Modeling glioblastoma heterogeneity as a dynamic network of cell states
Source: Mol Syst Biol. 2021 Sep 16;17(9):e10105. doi: 10.15252/msb.202010105 (PMC8444284; doi:10.15252/msb.202010105)
Supplement: Supplementary file 6 — Source Data for Figure 5 [file MSB-17-e10105-s004.zip › Figure5A_sourcedata/GSEA_3017/hallmarks_stateC.GseaPreranked.1621934520804/HALLMARK_P53_PATHWAY.html]

Details for gene set HALLMARK\_P53\_PATHWAY[GSEA]

|  || Dataset | state13017 |
| Phenotype | NoPhenotypeAvailable |
| Upregulated in class | na\_neg |
| GeneSet | HALLMARK\_P53\_PATHWAY |
| Enrichment Score (ES) | -0.28540286 |
| Normalized Enrichment Score (NES) | -1.1154963 |
| Nominal p-value | 0.3241491 |
| FDR q-value | 0.32349184 |
| FWER p-Value | 0.984 |
Table: GSEA Results Summary

  

Fig 1: Enrichment plot: HALLMARK\_P53\_PATHWAY      
 Profile of the Running ES Score & Positions of GeneSet Members on the Rank Ordered List

  

| PROBE | GENE SYMBOL | GENE\_TITLE | RANK IN GENE LIST | RANK METRIC SCORE | RUNNING ES | CORE ENRICHMENT || 1 | PCNA |  |  | 48 | 0.435 | -0.0105 | No |
| 2 | FBXW7 |  |  | 122 | 0.316 | -0.0839 | No |
| 3 | CCND2 |  |  | 149 | 0.288 | -0.0810 | No |
| 4 | UPP1 |  |  | 204 | -0.253 | -0.1320 | No |
| 5 | SAT1 |  |  | 241 | -0.262 | -0.1505 | No |
| 6 | PDGFA |  |  | 297 | -0.275 | -0.1997 | No |
| 7 | PPP1R15A |  |  | 342 | -0.293 | -0.2269 | No |
| 8 | FOS |  |  | 377 | -0.309 | -0.2343 | Yes |
| 9 | S100A4 |  |  | 395 | -0.318 | -0.2108 | Yes |
| 10 | GADD45A |  |  | 405 | -0.323 | -0.1727 | Yes |
| 11 | PTPRE |  |  | 469 | -0.372 | -0.2194 | Yes |
| 12 | HBEGF |  |  | 480 | -0.386 | -0.1726 | Yes |
| 13 | TSC22D1 |  |  | 492 | -0.400 | -0.1253 | Yes |
| 14 | JUN |  |  | 503 | -0.425 | -0.0720 | Yes |
| 15 | TM4SF1 |  |  | 580 | -0.689 | -0.0887 | Yes |
| 16 | S100A10 |  |  | 582 | -0.691 | 0.0241 | Yes |
Table: GSEA details [plain text format]

  

Fig 2: HALLMARK\_P53\_PATHWAY: Random ES distribution      
 Gene set null distribution of ES for **HALLMARK\_P53\_PATHWAY**
